# The role of daratumumab in relapsed/refractory CD38 positive acute leukemias—case report on three cases with a literature review

**DOI:** 10.3389/fonc.2023.1228481

**Published:** 2023-10-24

**Authors:** Witold Prejzner, Oliwia Piekoś, Karolina Bełdzińska, Alicja Sadowska-Klasa, Ewa Zarzycka, Maria Bieniaszewska, Krzysztof Lewandowski, Jan Maciej Zaucha

**Affiliations:** ^1^ Department of Hematology and Transplantology, Medical University of Gdansk, Gdańsk, Poland; ^2^ Department of Laboratory Medicine, Medical University of Gdansk, Gdańsk, Poland

**Keywords:** acute lymphoblastic leukemia, daratumumab, venetoclax, bortezomib, CD38

## Abstract

Primary refractory or relapsed T-cell acute lymphoblastic leukemia (T-ALL) and mixed phenotype myeloid/T-cell acute leukemia have dismal prognoses. New treatment approaches, preferably targeting specific leukemic aberrations to overcome resistance, are urgently needed. The bright expression of the CD38 antigen found in several cases of T-ALL led to an investigation into the role of anti-CD38 antibodies in the treatment of T-ALL. Here, we present three cases of resistant and relapsed T-ALL and myeloid/T-cell treated with daratumumab-based therapy, including venetoclax and bortezomib (Dara-Ven-Bor). All patients achieved complete remission, with minimal residual disease negativity within four weeks of treatment, allowing them to proceed to allogeneic hematopoietic cell transplantation. The toxicity of the triple schema was acceptable. Our patients and other cases reviewed here suggest that daratumumab combined with venetoclax and bortezomib may be a very effective and relatively safe salvage treatment, even in primary resistant T-ALL.

## Introduction

Primary resistant or relapsed (r/r) acute lymphoblastic leukemia have dismal prognoses. In particular, r/r T-cell acute lymphoblastic leukemias (T-ALL) have bleak prognoses, with approximately 10–20% long-term survival (1, 2). Similarly, mixed phenotype acute leukemia (MPAL) subtype myeloid/T-cell, a very rare leukemia subtype expressing both specific myeloid and T-cell antigens on leukemic cells, is often resistant to first-line treatment, which is not defined in this group of patients. Nelarabine used in monotherapy for r/r T-ALL achieves an overall response rate (ORR) of 50%, with a complete response (CR) rate of 36% (2). Patients that are resistant to nelarabine have very limited options, despite some anecdotal responses to different agents, such as dasatinib (3, 4) or venetoclax (5). Chimeric antigen receptor T-cell (CART cell) treatment is not available outside clinical trials. Therefore, the search for new drugs is ongoing, preferably targeting specific leukemic aberrations to overcome resistance. The challenge of developing such agents comes from the molecular heterogeneity of T-ALL blasts (6). Clinical trials in T-ALL have focused on therapies that inhibit Notch1 activation or cleavage of Notch proteins (bortezomib, crenigacestat) and BCL-2 inhibitors (venetoclax) (7–9). The expression of the CD38 antigen in leukemic cells prompted attempts to use monoclonal anti-CD38 antibodies (daratumumab, dara), which are very effective in patients with multiple myeloma (10). However, despite proven activity in xenograft models (11, 12), clinical experience with dara in r/r T-ALL is very limited. Monotherapy with dara was investigated in T-ALL patients with positive minimal residual disease (MRD) and a very good response was reported in published case reports (10, 13), which indirectly suggests its potential efficacy in a relapsed setting. Daratumumab combined with other agents targeting different mechanisms of leukemogenesis might potentially be more effective. Here, we present our experience in three cases of relapsed/refractory T-ALL and MPAL myeloid/T-cell patients with CD38 expression on leukemic blasts, treated with daratumumab-augmented salvage regimens.

## Case 1

A 20-year-old female with hyperleukocytosis 352 10^9^/l was diagnosed with T-ALL (patient characteristics, **Table 1**). Ninety percent of her bone marrow was infiltrated with blasts immunophenotyped as CD34+, CD33-, CD117-, CD19-, CD20-, CD3+, CD4-, CD5+, CD10+, TdT+, CD99+, CD79a+/-, CD7+, MPO -, CD38+, BCR : ABL1(-), KMT2A(-), karyotype 46,XX, and t (2, 14)(q21;q23)[5]/46,XX[7]]. She had no central nervous system (CNS) involvement. Initially, she received steroid pretreatment, followed by cyclophosphamide, with minimal leukocyte reduction. Subsequently, she immediately started a hyper-CVAD regimen (15) but without any meaningful response, with persistent blasts in peripheral blood. A salvage regimen consisting of nelarabine together with venetoclax and bortezomib (**Table 2**) was administered, taking into account primary resistance and planning for early haploidentical allogeneic transplantation. Moreover, daratumumab, with an interval of 14 days, was added, as the blasts were brightly CD38-positive. Intrathecal CNS prophylaxis was given (methotrexate 15 mg/ARA-C 40 mg dexamethasone 20 mg) twice during the Dara-Bor-Ven cycle. The toxicities were neutropenia grade 4 and nausea grade 2, according to the Common Terminology Criteria for Adverse Events. The patient achieved deep, complete remission with an MRD of 0.018% on day 28 and immediately underwent haploidentical hematopoietic cell transplantation (haplo-HCT) with a myeloablative total body irradiation (TBI)-based conditioning regimen. Engraftment was achieved on day 30. A bone marrow examination performed on the day of engraftment showed deep, complete remission with an MRD of 0.005% but with incomplete hematological recovery. She is alive with no signs of relapse 10 months after haplo-HCT, with full hematologic recovery and an MRD of 0.008%.

**Table 1:** Patient characteristics.

Pts	Organopathy	WBC (×10^9^/L)	Hgb (g/L)	Plt (×10^9^/L)	LDH (IU/L)	Blasts in PBS	Karyotype	Mutations	CD38+ expression)	Response to previous treatment
A.R.	Hepatosplenomegaly	351	13.3	124	1,316	89%	46,XX,t (2, 14)(q21;q23)[5]/46,XX[7]	FISH (-), NGS in progress	Positive Medium	Resistant
S.K.	Hepatomegaly	14	10.5	70		0%	46,XX,del (5)(q22q33),del (11)(q22q33)[12]/46,XY[9]	TRA/D (t14q11.2)	Positive Medium	Relapse
A.S.	Chloroma of right calf, hepatomegaly, lymphadenopathy	18	12.5	180	243	64%	No metaphases	None	Positive Bright (100%)	At diagnosis (2014)
A.S.	Relapse after two alloSCT	3.5	8.7	204	262	3,70%	No metaphases	None	Positive Medium	Before daratumumab

**Table 2:** Salvage therapies in three patients with CD38 positive acute leukemias.

Patient	Prior lines	Salvage treatment					MRD after salvage
		Nelarabine	Bortezomib	Venetoclax	Daratumumab	Peg-Asp	
Patient 1 A.R	1	1,500 mg/m^2^ 1,3,5	Twice a week (7 doses x 1 mg/m^2^) SC.	200 mg 1-28	16 mg/kg IV Every second week 3 doses	2,000 IU/m^2^ 7	0.018%
Patient 2 S.K	4	Resistant	Twice a week (8 doses x 1 m g/m^2^) SC.	200 mg 1-19	1,800 mg SC Every second week 3 doses	2,000 IU/m^2^ 7	0.024%
Patient 3 A.S	3	not given	Twice a week (4 doses x 1 mg/m^2^) SC.	100 mg 1-14	1,800mg SC Once a week 4 doses	NA	0.06%

## Case 2

A 24-year-old female was referred to our department as a war refugee from Ukraine, with a plan for a second allo-HCT for relapsed T-ALL (patient characteristics, **Table 1**). She was diagnosed with T-cell acute lymphoblastic leukemia (immunophenotypes CD33-, CD117-, CD19+/-, CD3+, CD4-, CD5+, CD10+, TdT+/-, CD99+, CD7+, MPO -, CD38+, and karyotype at diagnosis is unavailable) in November 2020. She received three cycles of the hyper-CVAD/MA protocol (15) but remained MRD-positive after reaching a complete hematological response. The patient underwent an allo-HCT from a mismatched unrelated donor after a conditioning regimen with treosulfan and fludarabine. She remained in CR but relapsed 8 months later. In Ukraine, she was offered salvage treatment consisting of methotrexate, vincristine, and doxorubicin, with no response. A third-line treatment consisting of clofarabine and cytarabine was given, but without a response. Subsequently, the fourth-line treatment (bortezomib with venetoclax followed by venetoclax monotherapy) was administered at a tertiary foreign center. The patient cleared the blasts in the bone marrow down to 2% but remained MRD-positive. At the time of her pre-transplant workup at our department, her bone marrow examination revealed that 3.56% of blasts (immunophenotypes CD34+, CD33-, CD117-, CD19-, CD3+, CD4-, CD5+, CD10+, TdT-, CD99+, MPO -, and CD38+), with the following karyotype: 46,XX,del (5)(q22q33),del (11)(q22q33)[12]/46,XY[9]; BCR : ABL1 and KMT2A were negative. Nelarabine (at a dose of 1,500 mg/m^2^, IV) with triple-agent prophylactic intrathecal chemotherapy was given as a bridge to haploidentical allo-HCT. Unfortunately, the disease progressed, with increased blasts of up to 54.12% in the bone marrow. As the sixth line of treatment, the patient received daratumumab, bortezomib, venetoclax, and PEG-asparaginase (**Table 2**). Intrathecal CNS prophylaxis (methotrexate 15 mg/ARA-C 40 mg dexamethasone 20 mg) was administered once during each Dara-Bor-Ven cycle. The observed toxicities included neutropenia grade 4, thrombocytopenia grade 4, anemia grade 3, and polyneuropathy grade 2. The patient achieved complete cytological remission with incomplete hematologic recovery (CRi) but with a deep MRD of 0.024%. She proceeded to the second allo-HCT from a haploidentical donor (mother) after a reduced toxicity conditioning regimen (TBI 8Gy/Flu). She was engrafted on day 24. A routine BM examination at day 28 showed normal, recovering bone marrow, with deep, complete remission (MRD 0.001%). The patient continued to remain in CR 10 months after transplantation, with a negative MRD (0.002%).

## Case 3

A 30-year-old woman was diagnosed with MPAL myeloid/T-cell in 2014 based on the immunophenotypes CD45+, CD34+, CD117+, HLA-DR+, cCD3+, CD3 (–), CD13+, CD7+, CD4+, cTdT+, CD99+, CD56+, and CD45RA+. BCR : ABL1 was negative, and the karyotype was unsuccessful (**Supplementary Table 1**). Notably, at diagnosis, she had an extramedullary lesion involving the calf. The patient received two cycles of hyper-CVAD/MA chemotherapy (15) augmented with L-asparaginase with intrathecal prophylaxis (**Supplementary Table 2**). After reaching CR, she underwent an allo-HCT from her matched brother after a full TBI/flu conditioning regimen. The patients developed severe chronic graft versus host disease (GvHD) (scleroderma) and required continuous immunosuppression (sirolimus, methotrexate, and extracorporeal photopheresis). She remained stable; however, 5 years later, she relapsed with extramedullary in the tonsils and local lymph nodes, without bone marrow involvement. She received nelarabine and achieved CR, as confirmed by positron emission tomography (PET). The patient deferred consolidation with a second allo-HCT from an alternative donor, preferring consolidation with local radiotherapy (**Supplementary Table 3**). She continued IST enhanced with ruxolitinib, which was reported to be effective in MPAL myeloid/T-cell (16). Three years later, she suffered from a second relapse, this time with bone marrow and CNS involvement, with many extramedullary lesions. Next-generation sequencing revealed mutations in CSF3R, WT1, ASXL1, and DDX41; the karyotype remained unknown due to a lack of mitotic activity. She was treated with azacitidine 75 mg/kg for 7 days and venetoclax (maximum dose 400 mg, decreased to 200 mg due to toxicity), with the addition of a high dose of cytarabine, followed by CNS irradiation. She reached her third CR and received a transplant from an unrelated donor after a thiotepa-based conditioning regimen (reduced TBF). Unfortunately, 2 months after the second transplant, she relapsed again. Salvage consisted of venetoclax for 14 days, bortezomib, and daratumumab because of CD38 positivity on leukemic cells (**Table 1**). A bone marrow examination at the end of the first cycle confirmed CR, with full hematological recovery and an MRD of 0.06%. Treatment was given to the outpatient with good tolerance, with no need for blood transfusion support. The patient developed some skin GVHD and remained leukemia-free with 100% donor chimerism for more than 9 months.

## Discussion

The rationale for adding daratumumab to different agents used as salvage chemotherapy for relapsed acute leukemias was the expression of CD38 on blast cells. CD38 (cyclic ADP ribose hydrolase) is a transmembrane glycoprotein expressed on the surface of plasma cells (12). CD38 is expressed on activated T-cells but is only expressed at low levels on normal lymphoid and myeloid cells and pluripotent hematopoietic stem cells, which makes treatment with CD38 antibodies safe for hematological toxicity (17). Recent studies have revealed that most T-ALL cells express CD38 antigens on the surface of blasts (14). Bride et al. demonstrated the presence of CD38 expression in T-ALL cells at diagnosis, after induction therapy, and at the time of relapse (11). The expression of CD38 in leukemic cells in our patients was strong (**Table 1**). CD38 expression in ALL patients is found in cases with T-cell phenotypes and was found to be positive in 97.9% of cases at diagnosis and 82.9% of refractory samples, as well as in B-ALL, where CD38 was present in 95.3% of B-ALL samples, with 88% positive in more than 70% of positive blasts (18, 19). We reviewed the phenotypes of our 61 consecutive patients. CD38 expression was present in all patients, including nine cases with T-cell phenotypes (three T-ALL and six MPAL/T myeloid patients) and 52 cases of B-ALL (**Supplementary Table 4**). The mechanism of action of daratumumab in CD38-positive acute leukemias is not clear; however, it might be similar to that in multiple myelomas. After ligation with the CD38 molecule, daratumumab inhibits tumor cell growth through complement-dependent cytotoxicity (CDC) and antibody-dependent cytotoxicity (ADCD) and induces cell apoptosis (20). In preclinical studies, daratumumab was proven to be a potential agent for the treatment of T-ALL, given its promising results in mouse models (11, 21). A preclinical study of pediatric T-ALL patient-derived xenografts found daratumumab to be effective in most patient-derived xenograft models, measured by a reduction in leukemic cell numbers (11). In another study, treatment with daratumumab eradicated MRD in seven of eight T-ALL xenograft mice (21).

The real-world data regarding the usage of daratumumab are limited to 34 cases (**Table 3**). Daratumumab has been used in pediatric and adult patients (24, 27). Daratumumab proved to be effective in patients with MRD positivity, especially those with MRD relapse after transplantation. Dara monotherapy led to long-lasting MRD-negative remission in three patients. The drug was started before overt hematological relapse (26). However, there is one case report of overt hematological relapse after two transplants with a long-term favorable outcome more than 3 years after starting single-agent daratumumab, having not responded to chemotherapy (dexamethasone, vincristine, pegylated L asparaginase, and bortezomib). After three doses of daratumumab, the patient became MRD-negative and achieved long-term remission for more than 3 years (22). The effectiveness of dara combined with chemotherapy was also shown in five patients with relapsed ALL. Four patients had T-ALL, one had B-ALL, and all patients were treated with daratumumab combined with or without chemotherapy. The number of daratumumab infusions was between 3 and 21, and 3 of 5 patients achieved CR (28). In addition, there is one case report of a patient who was resistant to standard chemotherapy and who was treated with salvage chemotherapy containing nelarabine, etoposide, and cyclophosphamide. After debulking, weekly daratumumab was started, and after three weekly doses of daratumumab, normal hematopoiesis was achieved in the bone marrow, and transplantation was performed (25). Recently, the largest population of patients treated with daratumumab was presented by an Italian group (13). The group presented data on daratumumab therapy from 20 patients who received at least one dose of daratumumab (dosage scheduled for multiple myeloma) between December 2018 and December 2020, with or without chemotherapy in ALL patients. Most patients (65%) were T-ALL, and the response rate for the whole group was 20% (n = 4). The median time to respond was 4 weeks. Three of the four responses were observed in patients with T-ALL who were treated with daratumumab monotherapy. The potential factors associated with a better response were ECOG performance status and fewer therapy lines (13).

**Table 3:** Real-world data of the usage of daratumumab in MRD positive and r/r/T-ALL.

Reference	No of pts.	Age (years/range)	Dgn	Relapse/primary resistant	CD38 expression	SCT before dara (Y/N)	Indication (MRD/treatment-relapse)	monotherapy/combined with chemotherapy	Response/time
(22)	1	26	ETP-ALL	3rd relapse	Bright	Y (2x)	Treatment	Mono	MRD neg/4 weeks
(10)	1	57	ETP-ALL	Primary resistant	97.7%	N	Treatment	Mono	CR/2 weeks MRD neg/8 weeks
(10)	1	26	ETP-ALL	MRD pos	Dim	Y	MRD pos	Mono	MRD neg/2 weeks
(23)	1	44	T-ALL	MRD pos (3%)	Unk	Y	MRD pos	Mono	MRD neg/2 weeks
(24)	1	38	T-ALL	1st relapse	98%	N	Treatment	Combined (nelarabine, Peg ASP, dexamethasone)	CR/3 weeks
(24)	1	25	T-ALL	Relapse after SCT	Strong	Y	Treatment	Combined (nelarabine, Peg ASP, dexamethasone)	CR 6 weeks/MRD 9 weeks
(25)	1	20	ETP-ALL	MRD pos after chemotherapy	Pos	N	MRD pos	Mono	MRD neg/3 weeks
(26)	1	24	T-ALL	MRD pos after chemotherapy for 3rd relapse	Pos	Y	MRD pos	Mono	MRD neg/8 weeks
(26)	1	44	T-ALL	MRD pos after chemotherapy for relapse	Pos	Y	MRD pos	Mono	MRD neg/4 weeks
(27)	1	2	T-ALL	Relapse	Pos	N	Treatment	Mono	PR/2 weeks
(28)	1	Unk	T-ALL	Relapse after SCT	Unk	Y	Treatment	Combined	CR
(28)	1	Unk	T-ALL	Relapse	Unk	N	Treatment	Combined	CR
(28)	1	Unk	T-ALL	Relapse	Unk	N	Treatment	Combined	
(28)	1	Unk	T-ALL	Relapsed after SCT	Unk	Y	Treatment	Mono	NR
(13)	20	8-73	13xT-ALL/4xB-ALL/1xMPAL/2xLBL	RR/MRD pos	Pos	9xY	Treatment/MRD	Mono/combined	20% CR/10% MRD neg

All our patients had primary refractory or relapsed CD38-positive acute leukemia. All were treated with a combined schema containing bortezomib, venetoclax, and daratumumab. One primary refractory case also received nelarabine. All three patients achieved remission with deep, negative MRD, including two patients with relapsed leukemia after prior allo-HCT. Two patients were successfully transplanted after achieving CR. The response was achieved very fast after 1 month of therapy, which is consistent with other reports (**Table 3**) (10, 23). We used dara combined with bortezomib and venetoclax, as both bortezomib and venetoclax were shown to be effective in T-ALL. We do not have data on Bcl-2 expression in our cases; however, in almost all ALL, BCL-2 expression was reported to be high (29). High Bcl-2 expression in T-ALL cell lines and high sensitivity to venetoclax-mediated BCL-2 inhibition were also reported in a preclinical study performed on T-ALL cell lines (29). Du et al. examined the cytotoxicity of bortezomib in combination with daunorubicin against human T-ALL cells. Bortezomib also enhances the cytotoxicity of ex vivo–expanded γδ T-cells against T-ALL cell lines (30). Preclinical data confirms the synergistic effect of dara combined with venetoclax, showing that antibody-dependent, cell-mediated NK cytotoxicity was enhanced in myeloma cell lines with a high expression of Bcl-2 (31). However, no data are available on leukemic cells. Therefore, it is difficult to assess the role of each agent in the treatment of our patients and whether dara monotherapy would be sufficient to achieve such a good response with surprisingly deep MRD negativity. The use of daratumumab in combination is a certain limitation, but it is hard to imagine that, in overt relapse, treatment with daratumumab monotherapy can be effective, and this is the reason we used a combined treatment. However, the fact that MRD negativity was achieved and the long duration of follow-up for the patient without relapse suggest the role of this drug, although our cases do not specify how big it was.

Combined therapy may increase toxicity, especially in heavily pretreated patients. However, our own experience and other reports suggest that dara with chemotherapy seems to be rather well tolerated and that there are few side effects; the most serious being neurological. Neurological side effects (described as maculopathy with paresis of the upper and lower limbs) were observed in one patient after transplantation with conditioning with TBI. The patient was treated with nelarabine and daratumumab; however, it is unclear which agent was responsible for the observed neurotoxicity (24). In fact, nelarabine in monotherapy is known to induce grade III–IV neurologic toxicities in approximately 10% of patients (2). Therefore, the combination of nelarabine and daratumumab should be used with caution. However, one patient was treated with nelarabine, and dara did not show any neurological toxicity.

All our patients were subjected to allo-HCT, including patient 3, who had severe chronic GVHD (cGVHD) after her first transplant. The effect of dara on the risk of immune complications is unknown, although its influence on immune composition, i.e., decreasing Treg cells, and an increase in T-helper cells and cytotoxic CD8+ T-cells upon dara treatment (32), may increase the risk of cGVHD. On the other hand, it has been suggested, based on murine models, that daratumumab may decrease the risk of cGVHD through multiple mechanisms, including inhibition of the proliferation, activation, and differentiation of CD8+ cytotoxic T-cells, reduced expression of cytotoxic effector molecules, pro-inflammatory cytokines, and promotion of immunosuppressive T-cells (33). The clinical findings suggest that dara treatment does not increase the risk of cGVHD. Nikolaenko et al. (34) did not observe an increased development of cGVHD in 34 relapsed multiple myeloma patients treated with dara after allo-HCT. Moreover, patient 3 did not experience worsened cGVHD after dara treatment.

We conclude that dara in monotherapy or combined with other agents may serve as an excellent salvage treatment, even in primary resistant ALL. Specifically, dara combined with bortezomib and venetoclax seems to be effective, with acceptable toxicity. Our cases encourage the start of clinical trials investigating the use of dara combined therapy in CD38+, R/R T-ALL, and MPAL myeloid/T-cells.

## Data availability statement

The original contributions presented in the study are included in the article/**Supplementary Material**. Further inquiries can be directed to the corresponding author.

## Ethics statement

The studies involving humans were approved by Komisja Bioetyczna ds. Badań Naukowych. The studies were conducted in accordance with the local legislation and institutional requirements. The participants provided their written informed consent to participate in this study. Written informed consent was obtained from the participants for the publication of any potentially identifiable images or data included in this article. 

## Author contributions

WP contributed to conception and design of the study, wrote draft of the manuscript OP, KB collected data, wrote sections of the manuscript AS-K collected data,wrote sections of the manuscript KL performed analysis, EZ collected data MB contributed to conception of the study JZ contributed to conception and design of the study, wrote draft of the manuscript All authors contributed to manuscript revision, read, and approved the submitted version.

## References

[1] Marks DI Paietta EM Moorman AV Richards SM Buck G DeWald G . T-cell acute lymphoblastic leukemia in adults: Clinical features, immunophenotype, cytogenetics, and outcome from the large randomized prospective trial (UKALL XII/ECOG 2993). Blood. (2009) 114(25):5136–45. doi: 10.1182/blood-2009-08-231217 PMC2792210 19828704

[2] Candoni A Lazzarotto D Ferrara F Curti A Lussana F Papayannidis C . Nelarabine as salvage therapy and bridge to allogeneic stem cell transplant in 118 adult patients with relapsed/refractory T-cell acute lymphoblastic leukemia/lymphoma. A CAMPUS ALL study. Am J Hematol (2020) 95(12):1466–72. doi: 10.1002/ajh.25957 32777149

[3] Frismantas V Dobay MP Rinaldi A Tchinda J Dunn SH Kunz J . Ex vivo drug response profiling detects recurrent sensitivity patterns in drug-resistant acute lymphoblastic leukemia. Blood (2017) 129(11):e26–37. doi: 10.1182/blood-2016-09-738070 PMC5356455 28122742

[4] Gocho Y Liu J Hu J Yang W Dharia NV Zhang J . Network-based systems pharmacology reveals heterogeneity in LCK and BCL2 signaling and therapeutic sensitivity of T-cell acute lymphoblastic leukemia. Nat Cancer (2021) 2(3):284–99. doi: 10.1038/s43018-020-00167-4 PMC8208590 34151288

[5] Richard-Carpentier G Jabbour E Short NJ Rausch CR Savoy JM Bose P . Clinical experience with venetoclax combined with chemotherapy for relapsed or refractory T-cell acute lymphoblastic leukemia. Clin Lymphoma Myeloma Leuk (2020) 20(4):212–8. doi: 10.1016/j.clml.2019.09.608 32035785

[6] Yadav BD Samuels AL Wells JE Sutton R Venn NC Bendak K . Heterogeneity in mechanisms of emergent resistance in pediatric T-cell acute lymphoblastic leukemia. 7:. ** Denotes Equal Senior Authorship. Oncotarget (2016) 7(37): 58728–58742. doi: 10.18632/oncotarget.11233 PMC5312271 27623214

[7] Lato MW Przysucha A Grosman S Zawitkowska J Lejman M . The new therapeutic strategies in pediatric t-cell acute lymphoblastic leukemia. Int J Mol Sci (2021) 22(9):4502. doi: 10.3390/ijms22094502 33925883 PMC8123476

[8] Borthakur G Martinelli G Raffoux E Chevallier P Chromik J Lithio A . Phase 1 study to evaluate Crenigacestat (LY3039478) in combination with dexamethasone in patients with T-cell acute lymphoblastic leukemia and lymphoma. Cancer. (2021) 127(3):372–80. doi: 10.1002/cncr.33188 33107983

[9] Sánchez-Martínez D Baroni ML Gutierrez-Agüera F Agüera A Roca-Ho H Blanch-Lombarte O . Fratricide-resistant CD1a-specific CAR T cells for the treatment of cortical T-cell acute lymphoblastic leukemia. Blood. (2019) 133(21):2291–2304. doi: 10.1182/blood-2018-10-882944 PMC6554538 30796021

[10] Mirgh S Ahmed R Agrawal N Khushoo V Garg A Francis S . Will Daratumumab be the next game changer in early thymic precursor-acute lymphoblastic leukaemia? Br J Haematol (2019) 187(2):e33–5. doi: 10.1111/bjh.16154 31452197

[11] Bride KL Vincent TL Im SY Aplenc R Barrett DM Carroll WL . Preclinical efficacy of daratumumab in T-cell acute lymphoblastic leukemia. Blood. (2018) 131(9):995–9. doi: 10.1182/blood-2017-07-794214 PMC5833263 29305553

[12] Wang X Yu X Li W Neeli P Liu M Li L . Expanding anti-CD38 immunotherapy for lymphoid Malignancies. J Exp Clin Cancer Res (2022) 41(1):1–18. doi: 10.1186/s13046-022-02421-2 35765110 PMC9237984

[13] Cerrano M Bonifacio M Olivi M Curti A Malagola M Dargenio M . Daratumumab with or without chemotherapy in relapsed and refractory acute lymphoblastic leukemia. A retrospective observational Campus ALL study. Haematologica. (2022) 107(4):996–9. doi: 10.3324/haematol.2021.279851 PMC8968887 35021604

[14] Jalal SD Al-Allawi NAS Al Doski AAS . Immunophenotypic aberrancies in acute lymphoblastic leukemia from 282 Iraqi patients. Int J Lab Hematol [Internet]. (2017) 39(6):625–32. doi: 10.1111/ijlh.12716 28722319

[15] Kantarjian HM O’brien S Smith TL Cortes J Giles FJ Beran M . Results of treatment with hyper-CVAD , a dose-intensive regimen, in adult acute lymphocytic leukemia. J Clin Oncol (2000) 18:547–61. doi: 10.1200/JCO.2000.18.3.547 10653870

[16] Verbeke D Gielen O Jacobs K Boeckx N De Keersmaecker K Maertens J . Ruxolitinib synergizes with dexamethasone for the treatment of T-cell acute lymphoblastic leukemia. HemaSphere (2019) 3:e310. Wolters Kluwer Health. doi: 10.1097/HS9.0000000000000310 31976483 PMC6924552

[17] Laubach JP Tai YT Richardson PG Anderson KC . Daratumumab granted breakthrough drug status. Expert Opin Investig Drugs (2014) 23(4):445–52. doi: 10.1517/13543784.2014.889681 24555809

[18] Tembhare PR Sriram H Khanka T Chatterjee G Panda D Ghogale S . Flow cytometric evaluation of CD38 expression levels in the newly diagnosed T-cell acute lymphoblastic leukemia and the effect of chemotherapy on its expression in measurable residual disease, refractory disease and relapsed disease: An implication for anti-CD38 immunotherapy. J Immunother Cancer (2020) 8(1):e630. doi: 10.1136/jitc-2020-000630 PMC7247386 32439800

[19] Eveillard M Floc’h V Robillard N Debord C Wuilleme S Garand R . CD38 expression in B-lineage acute lymphoblastic leukemia, a possible target for immunotherapy. Blood [Internet]. (2016) 128(22):5268. doi: 10.1182/blood.V128.22.5268.5268

[20] Overdijk MB Verploegen S Bögels M Van Egmond M Lammerts Van Bueren JJ Mutis T . Antibody-mediated phagocytosis contributes to the anti-tumor activity of the therapeutic antibody daratumumab in lymphoma and multiple myeloma. MAbs. (2015) 7(2):311–20. doi: 10.1080/19420862.2015.1007813 PMC4622648 25760767

[21] Vogiatzi F Winterberg D Lenk L Buchmann S Cario G Schrappe M . Daratumumab eradicates minimal residual disease in a preclinical model of pediatric T-cell acute lymphoblastic leukemia. Blood. (2019) 134(8):713–6. doi: 10.1182/blood.2019000904 31311816

[22] Punatar S Gokarn A Nayak L Bonda A Chichra A Mirgh S . Case Report Long term outcome of a patient with relapsed refractory early thymic precursor acute lymphoblastic leukemia treated with daratumumab. Am J Blood Res (2021) 11(5):528–33. doi: 10.1002/pbc.28829 PMC8610796 34824885

[23] Cerrano M Castella B Lia G Olivi M Faraci DG Butera S . Immunomodulatory and clinical effects of daratumumab in T-cell acute lymphoblastic leukaemia. Br J Haematol (2020) 191(1):e28–32. doi: 10.1155/2022/9722787 32686081

[24] Molle I Petruskevicius I Kamper P d’Amore F . Salvage therapy in early relapse of T-lymphoblastic leukemia/lymphoma using daratumumab/nelarabine combination: Two consecutive cases. Case Rep Hematol (2022) 2022:1–3. doi: 10.1038/s41375-019-0548-z PMC8763564 35047223

[25] Fulcher J Berardi P Christou G Villeneuve PJA Bredeson C Sabloff M . Nelarabine-containing regimen followed by daratumumab as an effective salvage therapy and bridge to allogeneic hematopoietic stem cell transplantation for primary refractory early T-cell precursor lymphoblastic leukemia. Leuk Lymphoma. (2021) 62(9):2295–7. doi: 10.1080/10428194.2021.1901097 33749497

[26] Ofran Y Ringelstein-Harlev S Slouzkey I Zuckerman T Yehudai-Ofir D Henig I . Daratumumab for eradication of minimal residual disease in high-risk advanced relapse of T-cell/CD19/CD22-negative acute lymphoblastic leukemia. Leukemia. (2020) 34(1):293–5. doi: 10.1002/pbc.29779 31435023

[27] Ruhayel SD Valvi S . Daratumumab in T-cell acute lymphoblastic leukaemia: A case report and review of the literature. Pediatr Blood Cancer. (2021) 68(5):1–2. doi: 10.1080/10428194.2021.1901097 33245179

[28] Vakrmanová B Nováková M Říha P Žaliová M Froňková E Mejstříková E . CD38: A target in relapsed/refractory acute lymphoblastic leukemia—Limitations in treatment and diagnostics. Pediatr Blood Cancer (2022) 69(9):2–5. doi: 10.1002/pbc.29779 35592935

[29] Peirs S Matthijssens F Goossens S Van De Walle I Ruggero K De Bock CE . ABT-199 mediated inhibition of BCL-2 as a novel therapeutic strategy in T-cell acute lymphoblastic leukemia. Blood. (2014) 124(25):3738–47. doi: 10.1182/blood-2014-05-574566 25301704

[30] Story JY Zoine JT Burnham RE Hamilton JAG Spencer HT Doering CB . Bortezomib enhances cytotoxicity of ex vivo-expanded gamma delta T cells against acute myeloid leukemia and T-cell acute lymphoblastic leukemia. Cytotherapy. (2021) 23(1):12–24. doi: 10.1016/j.jcyt.2020.09.010 33168453 PMC8075174

[31] Nakamura A Suzuki S Seto M Takasugi S Kanasugi J Hanamura I . Synergistic effect of venetoclax for antibody dependent cell cytotoxicity by daratumumab. Blood (2020) 136(Supplement 1):8–9. doi: 10.1182/blood-2020-134486 32614959

[32] Franssen LE van de Donk NW Emmelot ME Roeven MW Schaap N Dolstra H . The impact of circulating suppressor cells in multiple myeloma patients on clinical outcome of DLIs. Bone Marrow Transplant (2015) 50:822–8. doi: 10.1038/bmt.2015.48 25798669

[33] Gao Y Shan W Gu T Zhang J Wu Y Li X . Daratumumab Prevents Experimental Xenogeneic Graft-Versus-Host Disease by Skewing Proportions of T Cell Functional Subsets and Inhibiting T Cell Activation and Migration. Front Immunol. (2021) 12. 10.3389/fimmu.2021.785774 PMC8720868 34987512

[34] Nikolaenko L Chhabra S Biran N Chowdhury A Hari PN Krishnan A . Graft-versus-host disease in multiple myeloma patients treated with daratumumab after allogeneic transplantation. Clin Lymphoma Myeloma Leuk. (2020) 20(6):407–14. 10.1016/j.clml.2020.01.010 PMC9009296 32249196

