# Supplementary material for: The role of daratumumab in relapsed/refractory CD38 positive acute leukemias—case report on three cases with a literature review
Source: Front Oncol. 2023 Oct 24;13:1228481. doi: 10.3389/fonc.2023.1228481 (PMC10628456; doi:10.3389/fonc.2023.1228481)
Supplement: Supplementary file 1 [file DataSheet_1.pdf]

Supplementary

Table 1

The characteristics of genetic aberrations

Patient	#1	#2	#3
diagnosis			
	Pre-T ALL	T-ALL	MPAL T/myeloid
kariotype	46,XX,t(2;17)(q21;q23)[5] /46,XX[7].	No data	No methaphases
FISH	Not performed	No data	Not performed
mutation	none	TRA/D (t14q11.2)	none
immunophenotyp e	CD34+,CD33-, CD117-, CD19-, CD20-, CD3+, CD10+, TdT+, CD99+, CD79a+/-, CD7+,	No data	CD45+, CD34+, CD117+, HLA-DR+,cCD3+, CD3(-), CD13+, CD7+, CD4+, cTdT+, CD99+, CD56+, CD45RA+
Bcl-2 ekspression	Not performed	No data	Not performed
Before Dara-Bor-ven-Asp therapy			
kariotype	46,XX,t(2;17)(q21;q23)[5] /46,XX[7]	46,XX,del(5)(q22q33),del(11)(q22q33)[12]/46,XY[9]	No methapheses
mutation	none	none	*NGS -mutations in CSF3R, WT1, ASXL1, and DDX41*
immunophenotyp e	CD34+,CD33-, CD117-, CD19-, CD20-, CD3+, CD10+, TdT+, CD99+, CD79a+/-, CD7+,	CD34+,CD33-, CD117-, CD19-, CD3-, CD10+, TdT+/-, CD99+, CD79a+/- , CD7+, CD2 -, CD4-, , CD38+	CD45+, CD7+, CD117+, CD4-, CD123(var), CD56+, CD5-, cCD3+, CD2+, CD8-, CD33-, nTdT+, CD99+, sCD3-, TCRab-, CD44+, CD1a-, HLA-DR+, CD45RA-, TCRgd-, CD13+, CD38+
Bcl-2 ekspression	Not performed	Not performed	Not performed

Table2

Characteristic of patient’s previous therapy before DaraBorVenAsp therapy

Patient	#1	#2	#3
treatment	**First line**<br>hyperCVAD C1D1 18.07.2022 r.<br>response:<br>NR - MRD 41,71%<br>Toxicity -no<br>**Second line**<br>nelarabine (days 1,3,5), daratumumab (2, 15), bortezomib (twice a week - 5 x), peg-asparaginase (7 d.) C1D1 04.08.2022 r<br>venetoclax a la longue since 04.08.2022 r.<br>response<br>MRD – day 14 =>1,57%, day 21 =>0,05%, day 28. =>0,018%	**First line in Belarus**<br>3 x HyperCVAD/MA (21.11.2020-15.03.2021) - CR (MRD+)<br>TreoFlu + mMUD-alloPBSCT [9/10] (29.04.2021) - CR (MRD-) (05.2021)<br>**First. Relapse** (01.2022)<br>(**treated in Trukey**):<br>**1 line relapsed**<br>Rytuksymab + Metotreksat + WInkrystyna + Doksorubicyn (02-03.2022) - PD<br>**2 line relapsed** (Turkey)<br>Clofarabine + Cytarabine (04-05.2022) - PD (70% blasts)<br>**3 line**. **relapsed** (Turkey):<br>Bortezomib + Wenetoklaks since 06.2022) - CR (MRD <1%;) (28.06.2022)<br>**Maintanance**<br>Wenetoklaks monotherpy till 8.07.2022 - PD (MRD 3,564%) (11.07.2022)<br>**Second relapse**<br>.Gdansk, Poland<br>**1 line second relapse**<br>Nelarabina 1,5 g/m2 (26, 28, 30.07.2022) - PD (MRD 54,166%) (9.08.2022)<br>**2 line second relapse**<br>. Bortezomib + Daratumumab +	**First line**<br>2xHyperCVAD/MA+ Asparaginase=> CR<br>**Allo-PBSCT** (04.11.2014)<br>**First Relapse** (extramedullary – right tonsil and lymph nodes of neck 7.03.2019)<br>3x nelarabine (20.03 - 13.05.2019) - PET-CT (05.2019) – CR<br>+Radiotherapy of right tonsil+ ruxolitinib (07.2019-04.2022)<br>**2nd RELAPSE** (CNS involvement + extramedullary (skin, nasopharynx, kidneys, genitals, inguinal and iliac lymph nodes - 04.2022)<br>C1 Azacitidine 75mg/m2 (7days) + 4xAra-C 3000mg/m2 + venetoclax 400 mg x28 days<br>C2D1 25.05.2022 Azacitidine 75mg/m2 i Venetoclax 400-200-100mg - MRD(-)<br>**II Allo-PBSCT** (13.07.2022)<br>**3rd RELAPSE** (+80d II allo HCT - extramedullary disease 13.10.2022)

		Wenetoklaks + PEG-Asparaginaza (C1D1 - 12.08.2022) - iCR MRD 0,024% (6.09.2022)	C1D1 5.10.22 - Bortezomib (5.10,10.10, 13.10), Venetoclax, 05.10-20.10 -, Daratumumab - I 13.10, II 20.10. III 27.10.22, IV 4.11.2022<br><br>C2D1 14.11.2022 Azacitidine, Venetoclax, Daratumumab<br><br>C3 Daratumumab 12.01.23, Azacitidine+Venetoclax 16.01.23. + DLI - I dose 2.31 x 10^6 CD3/kg (27.1.2023)<br><br>C4 Azacitidine 100mg+Venetoclax 100mg 7 days (C4D1 20.3.2023)- II DLI 9.23x10^6 CD3/kg (29.3.2023)<br><br>⇨ CR , MRD (-)
alloSCT after Dara/Bor/Ven/Asp	Performed on 39 dy of second line cycle	Performed on42 days of the last cycle	N/A

Table 3

The characteristic of allo-SCT after Dara-Bor-Ven therapy (2 pts) and before Dara-Bor-Ven(1 pts)

Patient	#1	#2	#3
date	13.09.2022	26.09.22	13.07.2022
Type	haploidentical	haploidentical	MUD
Donor	father	mother	unrelated
Donor/Reciepent sex	M/F	F/F	M/F
HLA matching	7/10	5/10	10/10
conditioning	TBI 12 Gy (6-8.09.2022) Flu150 mg/m2 (10-12.09.2022)	TBI 8 Gy/ Fludarabine150 mg/m2	TBF
Source of stem cells	PBSC	PBSC	PBSC
immunosupression	PTCY/ MMF/ TAC	PTCy/MMF/TAC	ATG/CSA/MTX
GVHD	aGVHD late onset- skin overlapGVHD moderate skin -grade 2 - liver - grade. 1	aGVHD - no cGVHD - liver moderate grade2	aGVHD -no<br>cGVHD liver and skin grade 2
Response -MRD	+30 day - 0.005%<br>+100 day- 0.003%<br>+180 day- 0,008%	+30day- 0.001%<br>+100day- 0.002%<br>+180 days - 0.002%<br>Chimerism 100%,	+80 day relapse<br>Dara/bor/ven therapy

MUD- match unrelated donor , TBI -total body irradiation, TBF -Thiotepa Fludarabine Busulfan,

PBSC- peripheral blood stem cell, PTCY- post transplant cyclophosphamide, MMF –mycophenolate mofetil, TAC – tacrolimus, ATG antithymocyte globulin, CSA cyclosporine, MTX – methotrexate,

aGVHD -acute graft versus host disease , cGVHD -chronic graft versus host disease, MRD – minimal residual disease

Tabl 4. CD38 expression on blasts cell of B -ALL, T- ALL and MPAL myeloid/T cell

initials	Diagnosis	Date of Diagnosis	CD38 result
BN	pre-B-ALL	20.04.2022	positive med.
BJ	pro-B-ALL	01.02.2022	positive weak
FZ	common-B-ALL	02.12.2021	positive weak
GA	common-B-ALL	08.12.2021	positive weak
KA	MPAL T/My	2022-05-18	positive med.
KI	common-B-ALL	26.07.2022	positive med.
KD	common-B-ALL	17.09.2021	positive med.
LZ	common-B-ALL	22.12.2021	positive weak
LS	common-B-ALL	26.04.2022	positive weak
LA	common-B-ALL	30.08.2021	positive weak
MR	pre-T-ALL	07.02.2022	nd
MD	common-B-ALL	02.12.2021	positive weak
PA	pro-B-ALL	22.04.2022	positive weak
Pł	MPAL T/My	24.11.2021	positive med.
PA	common-B-ALL	11.05.2122	positive med.
RS	common-B-ALL	08.11.2021	positive med.
RA	pre-T-ALL	12.07.2022	positive med. (75%)
SA	MPAL T/My	01.04.2022	positive med.
SI	pro-B-ALL	02.08.2022	positive med.
SJ	common-B-ALL	30.11.2021	positive weak
SI	common-B-ALL	29.03.2022	neg./weak
SR	MPAL T/My	21.12.2021	positive med.
ZK	common-B-ALL	18.10.2021	positive med.
BS	MPAL T/My	13.12.2022	positive weak
BR	ETP	23.08.2022	nd
FY	common-B-ALL	04.11.2022	positive weak
HK	common-B-ALL	06.12.2022	positive weak
HR	common-B-ALL	28.09.2022	positive weak
JA	common-B-ALL	15.11.2022	positive weak
LD	pre-B-ALL	29.12.2022	positive weak
NP	pre-B-ALL	20.10.2022	positive med.
RA	common-B-ALL	25.10.2022	positive weak
SM	cortical T	16.11.2022	nd
M	cortical T-ALL	29.01.2021	nd
R	common-B-ALL	11.08.2021	positive med.
J	pro-B-ALL	01.03.2021	positive med.
I	pre-T-ALL	05.03.2021	nd
N	common-B-ALL	06.04.2021	positive med.
M	common-B-ALL	01.03.2021	positive med.
W	MPAL B/T	01.04.2021	positive med.

H	pre-B-ALL	14.05.2021	positive med.
Ł	cortical T-ALL	10.03.2021	nd
G	pre-T-ALL	08.03.2021	positive bright
M	common-B-ALL	06.04.2021	positive med.
M	common-B-ALL	30.04.2021	positive med.
D	common-B-ALL	13.04.2021	positive med.
K	common-B-ALL	18.03.2021	positive med.
M	common-B-ALL	07.06.2021	positive med.
M	pro-B-ALL	04.01.2021	positive med.
J	common-B-ALL	01.07.2021	positive weak
W	pre-B-ALL	24.06.2021	positive med.
P	common-B-ALL	15.02.2021	positive weak
B	common-B-ALL	12.05.2021	positive med.
K	mature-T-ALL	20.07.2021	positive bright
E	common-B-ALL	04.03.2020	positive med.
W	common-B-ALL	02.09.2020	positive med.
W	common-B-ALL	27.01.2020	positive med.
J	common-B-ALL	08.09.2020	positive med.
M	common-B-ALL	08.09.2020	positive med.
K	common-B-ALL	05.03.2020	positive med.
M	common-B-ALL	17.03.2020	neg/weak
B	pre-B-ALL	17.04.2020	positive weak
Wojciech	common-B-ALL	08.05.2020	positive weak
M	common-B-ALL	07.02.2020	positive bright
I	pro-B-ALL	05.03.2020	positive weak
B	common-B-ALL	30.04.2020	positive med.
S	common-B-ALL	27.01.2020	positive med.
M	pre-T-ALL	25.02.2020	nd
